# Quality of Work Life in Family Physicians of Bam, Iran 

**Published:** 2020-03

**Authors:** Akbarian Bafghi Akbarian Bafghi, Zahra Zare, Narges Rahimi

**Affiliations:** Department of Health Care Management, School of Public Health, Bam University of Medical Sciences, Bam, Iran

**Keywords:** Work Life Quality, Family Physician, Job Satisfaction

## Abstract

**Objective:** Quality of work life is one of the most important variables recently considered by many managers who seek to improve the quality of their human resources. Considering the vital factor of job satisfaction of family physicians as a service provider, this study was conducted to evaluate the quality of working life of family physicians in Bam.

**Materials and methods:** This research is a cross-sectional and descriptive type. Research population includes all family physicians working at health centers in Bam. The Van Larr Quality of Work Life Questionnaire, which was validated by Nekoei Moghadam, was used. The questionnaire data is analyzed by SPSS 24 from statistical-descriptive tests (mean and standard deviation) and T-test and ANOVA.

**Results:** Quality of work life score in family physicians is obtained 2.93 out of 5. Control ambiance in work (CAW) has the highest mean (3.16) and Work-life balance (WLB) has the lowest mean (2.29). Work experience has a meaningful relationship just with the two components of WLB and SAW in Quality of work life (p = 0.036). Furthermore, in the factor of GWB, the average score of unmarried physicians (Mean = 3.18) were more than the married ones (Mean = 3.05) (p = 0.010). The mean score of unmarried physicians was more than the married ones.

**Conclusion:** The quality of work life from the family physicians is about upper-intermediate. Thus, authorities of the family physician concept in the ministry of Health, and also in the Bam city should pay more attention to the family physician’s work life and consider programs and solutions in order to improve it.

## Introduction

Human resources are fundamental capitals of organizations, and the most important competitive advantage, and the scarcest resource in knowledge-centered economics. Also, they are counted as the source of every evolution and innovation in organizations. Therefore, the success and economic growth of every organization is depended on the effort and job satisfaction of that organization’s workers (1). Workers’ satisfaction is an important subject that can have a great influence over the amount of motivation. Therefore, job satisfaction includes the realities related to the salary, equipment control, and the organization’s functional terms (2) and can be determinative of quantity amount and future services quality (3). Job satisfaction causes increase organization’s performance and productivity, and the sense of satisfaction for individual. The job satisfaction is important because of the fact that most people spend their wakefulness in the work milieu (4). From the past up to now, job satisfaction investigating of workers, in order to increase their performance, has been considered by the health service organizations and their authorities who have used the results for solving organizational problems. Nowadays, a new concept of job satisfaction, as the work life quality is considered by the authorities (5). Work life quality, is a comprehensive and ambient program that causes increase in workers’ satisfaction and reinforcement in work milieu, and help workers to deal with changes and removals (6). The work life quality can be defined as the “relationship quality between workers and whole milieu’’ (7). This vital concept is in relation with workers’ consistency, resignation, organizational impression, productivity, life quality and etc. Therefore, there is world’s attention about the concept of work life quality (8). Job dissatisfaction can have negative effects on workers without paying attention to their job situation. Considering the importance of work life quality concept many authorities search for decreasing job dissatisfaction in all organizational levels and in management degrees (6). The domain of work life quality concept affects not only on the workers’ job dissatisfaction, but also on the out-of-work life of workers such as family, leisure, and social needs. When workers’ needs in work milieu are not fulfilled, it is possible that they do the job stressfully which would have unpleasant consequences on workers’ welfare and their working function (9).

The work life quality, initially was innovated in Europe and during the 1950s, and became formed on the basis of Eric Trist and his colleagues’ researches in university of Tavitak in the field of human relations in London (10). In recent two decades, health disciplines in developed countries and developing countries, based on unfulfilling the health needs and expectations, and also based on performing the reforming programs, are under pressure. Without a doubt, health discipline of Iran, in spite of various and worthy achievements that have reached in recent decades, has many challenges and issues. Reforms in Iran’s health discipline are inevitable because of many reasons (11). The aim of reforming the health discipline in Iran is starting a hierarchy of purposeful and continuous changes for amending the performance, commission of justice for people privilege of health services, protecting people from financial dangers of diseases, constant financial support, and reforming the payment system. One of the works which has been done in this field, is compiling the regulation of referring system and family physician (12).

Based on the budget in 2016, the health insurance organization was obliged to provide the enjoyment of health services in format of family physician program and referring system, by issuing medical insurance services for all rural and urban residents with a population under 20000 (13).

Performing the concept of rural insurance is done through the focus of family physician and referring system, with the purpose of invention and refining the referring system in the country, accountability increasing in health market, increasing people access to health services, and increase in services covering (14). In the family physician program and referring system as one of reforming programs in health system, general practitioner and his team have the whole responsibility over the individuals’ health and the families who are covered by them, and after referring the individual to specialized level, they have the responsibility for following their fate (15). Satisfaction of service providers has direct impression on service receivers, therefore the job satisfaction of family physicians is an important factor for health systems (16). The physician’s dissatisfaction is related to reducing loyalty for curing the patient, worsening the quality of received cares, prescriptive errors and increasing the amount of medical errors that endangers the patient’s safety (17).

Many studies state the dissatisfaction of service providers and receivers, especially the family physicians from the family physician program. For example, in the case study of Ebadifar Azar and colleagues, only 46 percent of patients had satisfaction from family physician program, on the other side, family physicians themselves as the service providers have expectations from their job, that if the suitable response is not found for them, it may result the dissatisfaction in both service providers and receivers in health system (18). In the case study that was done in England during 90s _1987_ and also continued until 1998, it was shown that the degree of satisfaction in male and female physicians was reduced from 1987 until 1990 and then it was increased until 1998 (19). During another case study which was done in USA in 1996-97 for determining general practitioners’ and family physicians’ job satisfaction, it was shown that the amount of job dissatisfaction for general practitioners was 17 percent and for family physicians it was 20 percent (20). Researchers consider various factors such as unsuitable payment circumstances and salary, much working hours, exceeded paperwork, missing professional job independency, less consistency of job and continuing education, maltreatment of people, milieu-related issue, and much patient referral, all important for their less permanency and dissatisfaction (21). Based on the study which was done by Van laar and his colleagues, in order to determine the scale for measuring quality of work life in health workers in 2007, the 6 indexes below are specified as the most important impressive factors over the quality of work life:

1- Career job satisfaction (CJS)

2- Work conditions (WCS)

3- General well-being (GWB)

4- Work-life balance (WLB)

5- Stress at work (SAW)

6- Control at work (CAW)

The attention which is paid nowadays to the quality of work life, is reflection of the importance which people are aware of this topic. According to the research done by Oswald 2002 in USA, job satisfaction in English workers was 49%, in Denmark 62%, in Japan 30%, and in Hungary it was 23% (22). 

Since the performance of family physician concept is a fundamental action and an important evolution in the case of providing medical health care and referring system in Iran, and considering the importance of job satisfaction factor and family physicians as the service provider in primary level, and the importance of primary-level health care system due to the its relativity with large population, and the topic of covering all individuals of a society in order to provide hygiene services, the aim of this research is determining the amount of quality of work life expressed by family physicians, and quality of work life in order to upgrade their level of performance and increasing the covered people satisfaction.

## Materials and methods

This study was a descriptive and applied type and was done cross-sectional in 2017 in health centers of the Bam. Ethics Code of this paper is MUBAM.REC.1395.17. The research population of this study includes all family physicians working at medical health care centers in the Bam in 2017. For data gathering the questionnaire which was designed by Ovan Laar and his colleagues with the title “health workers’ quality of work life scale” was used (23). Also, the stability of the mentioned questionnaire was measure by Cronbach’s alpha coefficient which was 0.815. This questionnaire had high stability, and also for validity verification, this questionnaire was verified by some masters and clear-sighted people from management group, and the needed reforms were used.

Also, the stability of the mentioned questionnaire measured by open test by Dr. Nekouei Moghaddam and his colleagues in an article titled “Investigating family physicians’ quality of work life in Kerman province” (5) and also the study of Shabani Nezhad and colleagues in Mazandaran province titled “Investigating quality of work life expressed to family physicians” (6), in which there was correlation coefficient about 95% between questions. Cronbach’s alpha for measuring internal relation of questions was 78%, and with segregation of questions, the before and after correlation coefficient was between 1 to 54 percent. 

The mentioned questionnaire is regulated in two parts: The first part includes information about the demographic status of family physicians, and second part includes questions which are derived from CJS factors like “I’m satisfied from my life”, “Most of the times my life is near to my ideals”, “Generally everything goes fine for me”, “I’m satisfied from the job opportunities made in my work place”, “Recently, by considering all conditions I feel nice”, “Totally, I have satisfaction from my quality of work life”. WCS which includes questions which are: “I have the opportunity for using my whole potential in work place”, “When I do something well, I became encouraged and admired by my supervisor”, “I’m encouraged to learn new skills”, “My employer provides all I need to do my job efficiently”, “My supervisor manager provides flexible working time”, “I work at a safe milieu”, “I feel satisfied from the instructions I get for doing my job better”, “My working conditions are satisfying”. GWB includes questions such as “Right now I feel well”, “Recently I had feeling of depression and inconvenience”. WLB includes question like “My employer provides me enough flexibility and facilities to make it possible for me to have balance between my working and family life”, “My recent working hours fit my personal life”. SAW includes questions: “Often, I feel I’m under pressure in my work place”, “Most of the times, I feel intensive pressure in my work place”. CAW includes questions “I have marked working aims which provide me abilities for doing the job”, “I feel I can express my comments and I have impression over my work place changes”,

“I’m contributed in the decisions made in my work place which affect me”, “I’m contributed in the decisions made on the people who are impressive in my work place”, that measures physicians’ quality of work life. The scale which is used in this questionnaire is the Likert scale. Each one of these questions in the specter of (1-5 for Strongly Disagree-Disagree-Neutral-Agree-Very Agree) is evaluated and aggregated. Analysis of gathered data by the usage of descriptive statistics including mean and standard deviation, and in order to compare the life quality mean in various groups of independent t-test and one-sided variance analysis, and other related tests by the help of SPSS software Version 24 is used.

## Results

Among 53 family physicians in this case study: about 75.5% (40) of them were 25 to 30 years old. about 45.3 (24) of them were male and 54.7% (29) were female. Also, 83% (44) of physicians have experience over 3 years. almost 50.9% (27) of family physicians were unmarried and 40.1% (26) of them were married. Most number of physicians 62.3% (33) have salary between 50 to 100 million Rials.

About 41.5% (22) of physicians were formal recruitment, 50.9% (27) of them were contractual, and finally 42.5% (22) of physicians were residing in Bam.

According to results of the study between different scopes from quality of work life, CAW had the highest mean (3.16 percent), and WLB has the lowest mean (2.29), and the mean of total QWL is 2.93 out of 5 (Table 1).

According to the ANOVA test there is no significant relationship among the age, salary, type of employment, work place, work experience with total score from quality of work life. 

Work experience variable had meaningful relationship with SAW (p = 0.036).

T-test results for determining relationship between sex and marriage status with quality of work life total score, stated that had not meaningful relationship between them but showed that there is meaningful relationship between marriage status and the factor of GWB (p = 0.010), the average score of unmarried physicians (Mean = 3.18) were more than the married ones (Mean = 3.05). 

There is no relationship between each dimension of quality of work life and sex, age, salary, type of employment, work place, work experience.

## Discussion

This study was done in order to investigate the quality of work life among family physicians in Bam. Most of the investigated people were female and unmarried, and also most of them were contractual physicians who had experience over 3 years.

Just as the results of this study stated, quality of work life among physicians in Bam is at intermediate and high level. Since the low quality of work life may influence over quality and undertaking of services and be an impressive factor related to the shortage of health service providing (24), thus, the authorities of family physician concept in Ministry of Health and also the authorities of this concept in Bam must pay more attention to the quality of work life among family physicians, and should consider programs and solutions for upgrading this. 

Based on reviewing medical magazines and articles related to this topic in other countries, there is a general agreement which states that job satisfaction among family physicians is reducing. Population growth and patients’ expectations, increasing time pressure and payment limitation, are all the factors which impress over physicians’ job dissatisfaction (24).

It seems that low life quality among physicians causes reducing productivity and has negative effect over service providing, and may cause the failure of family physician concept. Paying attention to family physicians’ quality of work life can result in amending service providing in health system and the patient’s sense of satisfaction which is the goal of every health system. Results of this study showed there is no meaningful difference among the quality of work life from female and male family physicians. These findings correspond with the studies of Arab and colleagues (24), Eker and colleagues (25), and Shabani Nezhad (6). 

According to the mentioned investigations, quality of work life among unmarried family physicians in the factor of GWB is more than the marrieds, and states that not working at their residential location brings problems for married family physicians, and conclusively affects their quality of work life, because generally the life is temporary possible in service-providing location for unmarried family physicians, and it may not be possible for married physicians.

**Table 1:** Comparing family physicians’ quality of work life in Bam in different scopes

**Scopes of QWL** ^1^	**CAW** ^2^	**GWB** ^3^	**SAW** ^4^	**CJS** ^5^	**WCS** ^6^	**WLB** ^7^	**QWL**
Mean score (1-5 scale)	3.16 ± 0.94	3.11 ± 0.50	3.00 ± 1.01	2.90 ± 0.59	2.89 ± 0.85	2.29 ± 0.92	2.93 ± 0.46

1Quality of working-life, 2Control at work, 3General well-being, 4Stress at work, 5Career and job satisfaction, 6Work conditions, 7Work-life balanc

In this study, quality of work life mean is calculated through 6 scopes, and through the study data, CAW scope has the highest mean 3.16 and WLB has the lowest mean 2.29 out of 5, and it happens while in other researches like the study of Arab and colleagues (24), and Nekoei Moghaddam and colleagues (5), GWB has the highest mean. In the study of Arab and colleagues (24) WLB has the lowest mean, and in the study of Nekouei Moghaddam and colleagues (5) the scope of CAW has the lowest mean.

In the study of Shabani Nezhad and colleagues (6) the scope of CAW has the highest mean, and WLB has the lowest one. Probably, the difference among means in the mentioned scopes is because of the difference in provinces and equipments, motivation, stress, and the conditions dominating the case study environment. According to the case study results, attention to the quality of work life can cause the sense of interdependency, responsibility, and finally increasing the productivity, performance and impression of family physicians’ function. Case study results of Dehghan Nayeri (26) shows that only 0.1 of the investigated sample have reported their quality of work life in a good state.

Also, in the study done by Smith (10) about the quality of work life among nurses in USA, the same results were achieved. Since the study results show that among the quality of work life factors, WLB had the lowest measure, so it’s needed that for more harmonizing between quality of work life and personal life of family physicians, for the goal of upgrading quality of work life level, some actions should be done.

The concept of family physician initially had many problems such as job dissatisfaction, financial disturbances, lack of time and patience for examining and etc. it seems it’s a long time that enough emphasis is not considered about this issue by the health system of the country. This is while that most of the physicians do their best in order to eliminate these problems, and senior politicians of health system can cause amendment in service providing state of physicians and solving their function by upgrading quality of work life in various dimensions such as job satisfaction, job conditions, GWB, WLB, SAW, and CAW.

## Conclusion

The quality of work life from the family physicians is intermediate and upper-intermediate. Thus, authorities of the family physician concept in the ministry of Health, and also in the Bam city should pay more attention to the family physician’s work life, and consider programs and solutions in order to improve it. Also If planning for family physicians be accurate and correctly, it can make sense of dependence, responsibility and thus increase productivity, efficiency and effectiveness of family physician’s performance.
